# Up regulation of the Hippo signalling effector YAP1 is linked to early biochemical recurrence in prostate cancers

**DOI:** 10.1038/s41598-020-65772-w

**Published:** 2020-06-02

**Authors:** Andreas Marx, Aljoscha Schumann, Doris Höflmayer, Elena Bady, Claudia Hube-Magg, Katharina Möller, Maria Christina Tsourlakis, Stefan Steurer, Franziska Büscheck, Till Eichenauer, Till S. Clauditz, Markus Graefen, Ronald Simon, Guido Sauter, Jakob R. Izbicki, Hartwig Huland, Hans Heinzer, Alexander Haese, Thorsten Schlomm, Christian Bernreuther, Patrick Lebok, Adam Polonski

**Affiliations:** 1 Institute of Pathology, University Medical Centre Hamburg-Eppendorf, Hamburg, Germany; 2 Institute of Pathology, Klinikum Fürth, Fürth, Germany; 3 General, Visceral and Thoracic Surgery Department and Clinic, University Medical Centre Hamburg-Eppendorf, Hamburg, Germany; 4 Martini-Clinic, Prostate Cancer Centre, University Medical Centre Hamburg-Eppendorf, Hamburg, Germany; 5 Department of Urology, Charité - Universitätsmedizin Berlin, Berlin, Germany; 6 Department of Urology, University Medical Centre Hamburg-Eppendorf, Hamburg, Germany

**Keywords:** Prognostic markers, Prostate

## Abstract

The transcriptional coactivator YAP1 controls the balance between cell proliferation and apoptosis. YAP1 overexpression is linked to poor prognosis in many cancer types, yet its role in prostate cancer is unknown. Here, we applied YAP1 immunohistochemistry to a tissue microarray containing 17,747 clinical prostate cancer specimens. Cytoplasmic and nuclear YAP1 staining was seen in 81% and 63% of tumours. For both cytoplasmic and nuclear YAP1 staining, high levels were associated with advanced tumour stage, classical and quantitative Gleason grade, positive nodal stage, positive surgical margin, high KI67 labelling index, and early biochemical recurrence (p < 0.0001 each). The prognostic role of YAP1 staining was independent of established prognostic features in multivariate models (p < 0.001). Comparison with previously studied molecular markers identified associations between high YAP1 staining, *TMPRSS2:ER*G fusion (p < 0.0001), high androgen receptor (AR) expression (p < 0.0001), high Ki67 labelling index (p < 0.0001), and *PTEN* and 8p deletions (p < 0.0001 each). In conclusion, high YAP1 protein expression is an independent predictor of unfavourable disease course in prostate cancer. That cytoplasmic and nuclear YAP1 staining is equally linked to phenotype and prognosis fits well to a model where YAP1 activation during tumour progression includes up regulation, cytoplasmic accumulation and subsequent translocation to the nucleus.

## Introduction

In 2018, prostate cancer was the most common cancer in males and the third most cause of cancer related death^1^ with more than 1.3 million estimated newly diagnosed cases worldwide. The clinical course is variable and the currently used criteria for the distinction between high risk and low risk patients are Gleason grade, clinical stage and PSA value. To further reduce overtreatment, molecular prognostic markers would be an advance.

The transcriptional coactivator YAP1 is the critical downstream regulator of the Hippo signalling pathway that controls the balance between cell proliferation and apoptosis during embryogenesis and organ development^2,3^. Phosphorylation of cytoplasmic YAP1 and/or its paralogue WWTR1 by kinases of the Hippo pathway inhibits YAP1’s translocation to the nucleus where it activates target genes important for cell proliferation, cell death and cell motility^4,5^. Recent studies highlight a critical role of Hippo-YAP1 signalling for the biology of a wide range of cancer types. For example, in more than 90 studies published in Pubmed as to yet (March 2020), up regulation of YAP1 was reported from cancers of the cervix^6^, endometrium^7^, oesophagus^8^, urinary bladder^9^, brain^10,11^, skin^12^, head and neck^13^, ovary^14^, mesothelium^15^, bones^16^, lung^17^, breast^18^, colon^19^, stomach^20^, pancreas^21^ and liver^22^, and was linked to adverse tumour features and/or poor patient prognosis in most tumour types.

There is growing evidence that YAP1 migth also play an important role for the biology of both early and late stage prostate cancers. *In vitro* models suggest that YAP1 induces growth and migration in normal prostate epithelial cells^5^, revealed functional relationships between YAP1 activity and the prostate cancer specific TMPRSS2:ERG gene fusion^23^ as well as the PTEN tumour suppressor^24^, which is lost in about 20% of prostate cancers^25^, and that interaction of YAP1 with the androgen receptor may contribute to the development of castration-resistant prostate cancer^26^. Although these findings make YAP1 a promising candidate for a useful clinical marker in prostate cancer, five validation studies applying immunohistochemistry to 20–188 prostate cancers reported inconclusive results: There was either reduced^27,28^, unchanged^29^ or up regulated^5,30^ YAP1 in tumours as compared to normal or benign prostate tissues. Also, both high^28,29^ and low^27^ YAP1 protein levels have been reported to be linked with unfavourable tumour phenotype.

This study was undertaken to better understand the role of YAP1 in clinical prostate cancer samples. Here, we employed YAP1 immunohistochemistry (IHC) in a tissue microarray containing more than 14,000 prostate cancers with clinical follow-up data.

## Results

### Technical issues

A total of 9,571 (69%) and 9,884 (71%) tumour samples were interpretable for cytoplasmic and nuclear staining in our TMA analysis. The remaining tumors were considered non-informative because they either lacked unequivocal cancer tissue in the 0.6 mm spot or the entire tissue spot was missing on the TMA section.

### YAP1 expression in normal and cancerous glands

**Figure 1:**
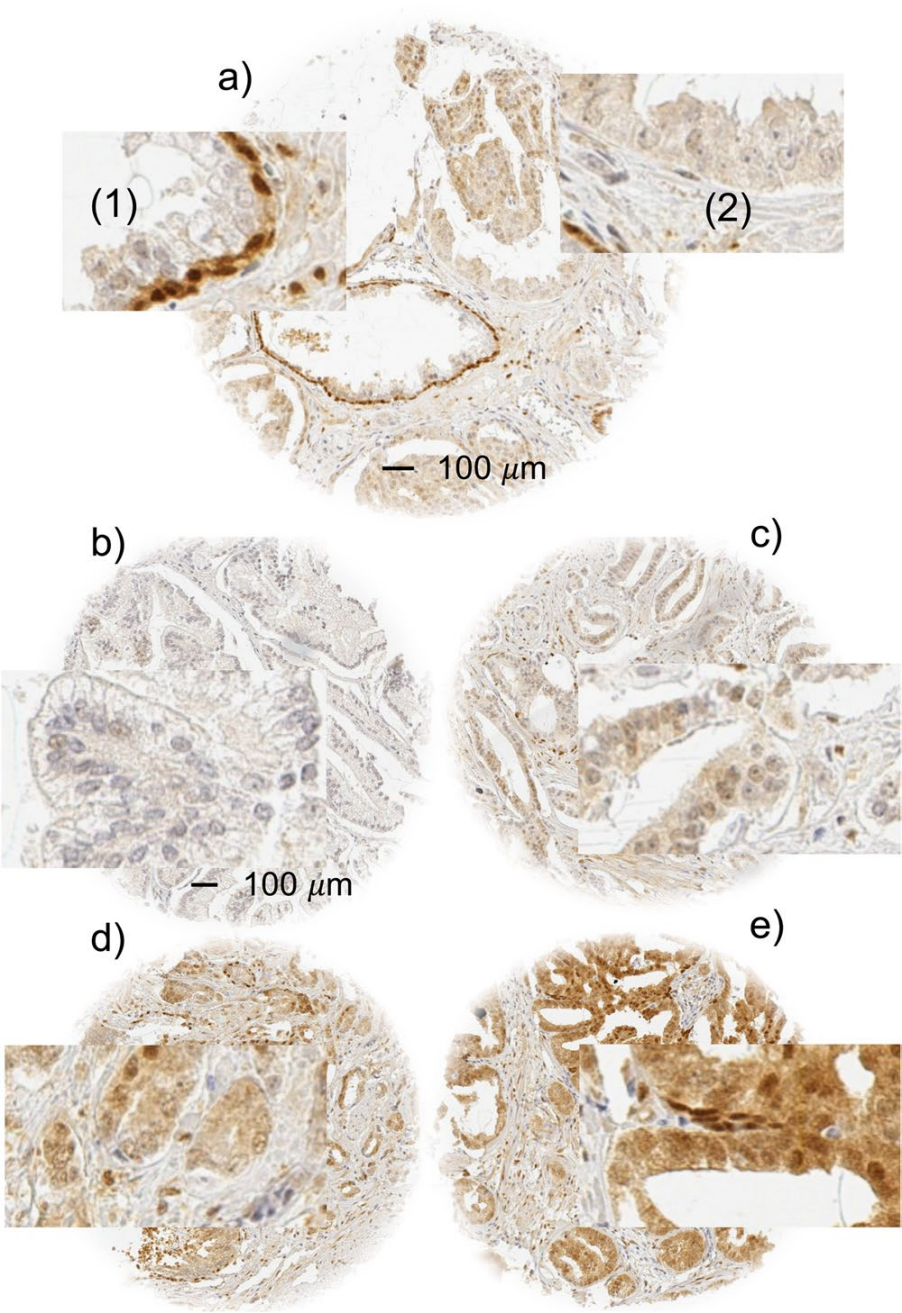
Examples of YAP1 staining in prostate tissue. (**a**) 0.6 mm tissue spot with normal and cancerous glands. Insets show strong YAP1 staining in (1) basal cells of the normal glands but absence of detectable staining in luminal cells (2) of tumour glands. (**b–e**) shows example of cancers with negative (**b**), weak (**c**), moderate (**d**) and strong (**e**) YAP1 staining.

**Figure 2:**
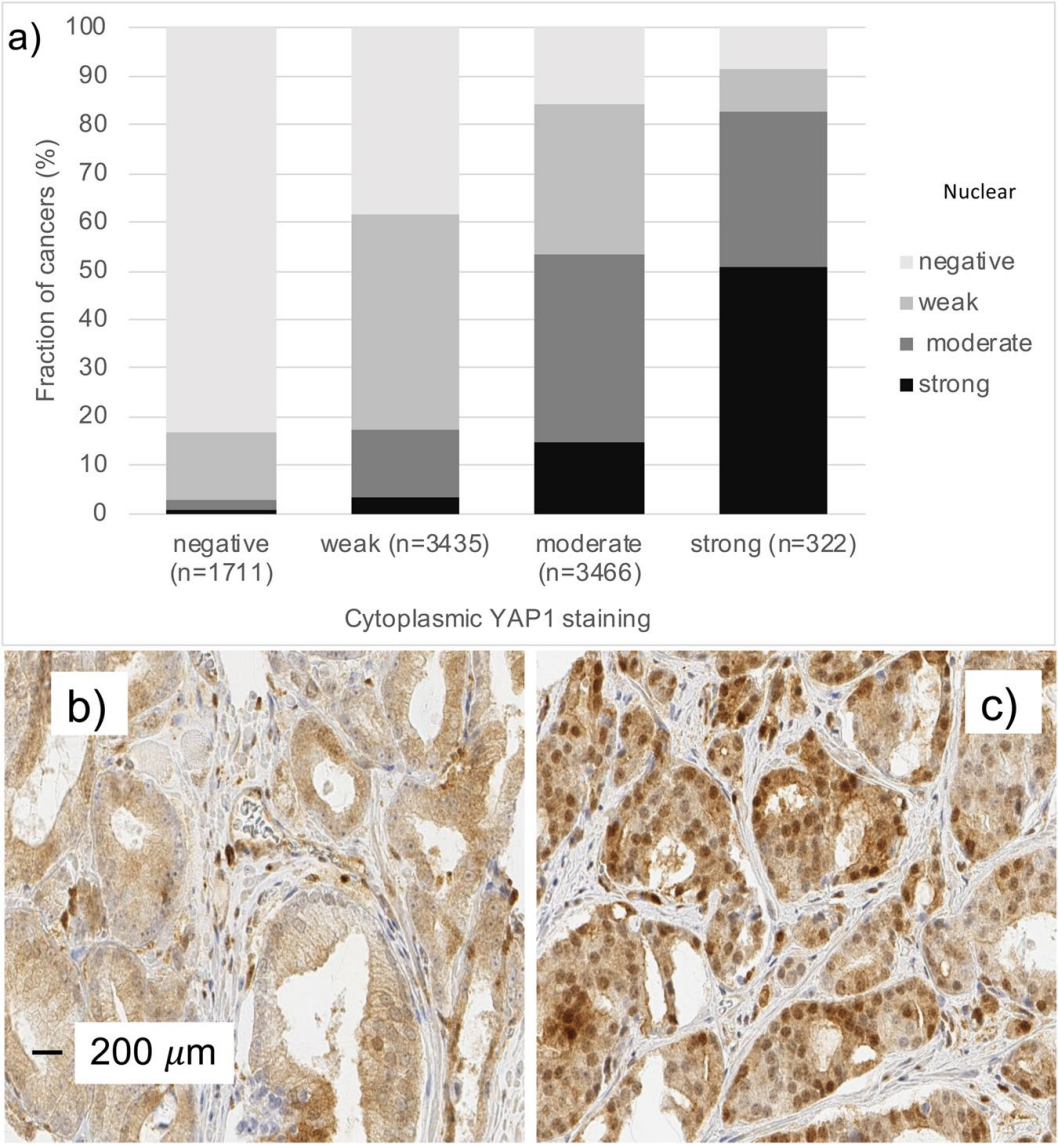
Cytoplasmic and nuclear YAP1 staining. (**a**) Significant correlation between cytoplasmic and nuclear YAP1 (p < 0.0001). (**b**) Example of a cancer with purely cytoplasmic YAP1 staining. (**c**) Example of a cancer with cytoplasmic and nuclear co-expression of YAP1.

**Figure 3:**
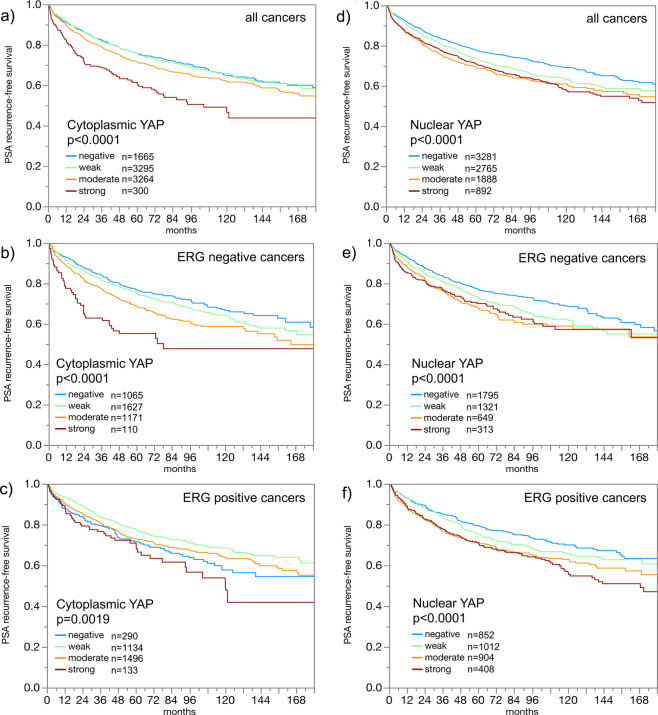
Prognostic role of YAP1 in prostate cancer. (**a–c)** Impact of cytoplasmic staining (irrespective of nuclear staining) on PSA recurrence-free survival in (**a**) all cancers, (**b**) ERG negative cancers and (**c**) ERG positive cancers. (**d–f)**: Impact of nuclear staining (irrespective of cytoplasmic staining) on PSA recurrence-free survival in (**d**) all cancers, **(e**) ERG negative cancers and (**f**) ERG positive cancers.

Normal prostatic glandular cells showed variable levels of cytoplasmic and nuclear staining ranging from negative to moderately positive, while basal cells always showed strong nuclear and often also cytoplasmic staining. In prostate cancers, cytoplasmic and nuclear staining was seen in 80.9% and 62.9% of tumours and was considered weak in 39%/32% (cytoplasmic/nuclear), moderate in 39%/22%, and strong in 4%/10% of cancers. Examples of cytoplasmic and nuclear YAP1 immunostainings in normal prostate and prostate cancers are shown in Figs. 1 and 2a,b. Cytoplasmic and nuclear staining was strongly linked to each other. For example, only 1% of 1,711 cancers with negative cytoplasmic staining, but 51% of 322 tumours with strong cytoplasmic staining showed strong nuclear staining (p < 0.0001, Fig. 2c). Both increased cytoplasmic and increased nuclear YAP1 staining were significantly linked to high traditional and quantitative Gleason grade (p < 0.0001), high pT category (p < 0.0001), nodal metastasis (p ≤ 0.03, Table 1, Supplementary Table S1), and early biochemical recurrence (p < 0.0001 each, Fig. 3a,b). Examples of YAP1 immunostaining in cancers with different Gleason grades are shown in Supplementary Fig. S1.

**Table 1:** Cytoplasmic YAP1 staining and prostate cancer phenotype.

	**Cytoplasmic YAP1 staining (%)**					
	**N**	**Negative**	**Weak**	**Moderate**	**Strong**	***P***
**All cancers**	9571	19.1	38.6	38.7	3.5	
**Tumour stage**						<0.0001
pT2	5967	20.2	40.1	37.1	2.5	
pT3a	2261	17.4	37.0	40.9	4.8	
pT3b-pT4	1308	17.0	34.6	42.7	5.7	
**Gleason grade**						<0.0001
≤3 + 3	1775	27.0	41.0	30.1	1.9	
3 + 4	5171	18.1	38.6	39.9	3.4	
3 + 4 Tert.5	440	17.0	39.8	42.0	1.1	
4 + 3	987	15.3	37.5	42.5	4.8	
4 + 3 Tert.5	679	12.8	38.7	42.6	5.9	
≥4 + 4	512	19.7	32.2	41.4	6.6	
3 + 4 ≤ 5%	1292	20.2	39.2	38.1	2.5	<0.0001
3 + 4 6–10%	1347	17.1	39.7	39.9	3.3	
3 + 4 11–20%	1171	18.6	36.6	41.4	3.3	
3 + 4 21–30%	608	15.6	40.3	40.6	3.5	
3 + 4 31–49%	520	17.1	36.2	41.3	5.4	
4 + 3 50–60%	419	16.0	38.2	42.5	3.3	
4 + 3 61–80%	375	13.6	38.1	42.1	6.1	
4 + 3 > 80%	96	12.5	28.1	54.2	5.2	
**Lymph node metastasis**						0.0237
N0	5656	17.4	38.5	40.2	3.9	
N +	648	18.2	32.6	44.3	4.9	
**Preoperative PSA level (ng/ml)**						<0.0001
<4	1104	14.8	36.9	43.7	4.7	
4–10	5714	18.4	38.8	39.5	3.3	
10–20	1980	22.3	40.1	34.3	3.3	
>20	709	23.4	36.5	36.2	3.8	
**Surgical margin**						0.4865
Negative	7522	19.1	38.8	38.7	3.4	
Positive	2011	19.3	37.8	38.9	4.0

### YAP1 and *TMPRSS2:ERG* fusion status

**Figure 4:**
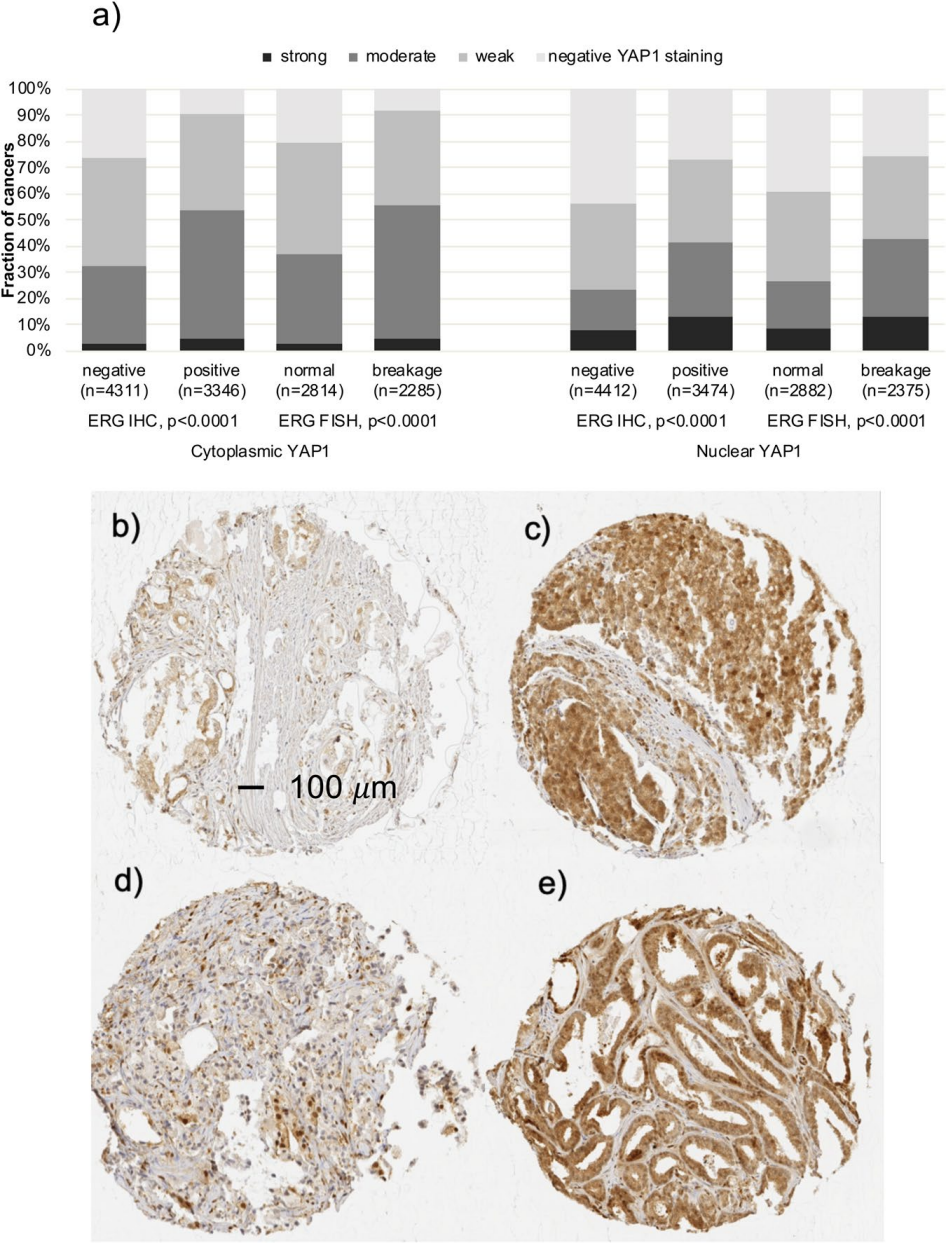
YAP1 and ERG. (**a**) Correlation between cytoplasmic (left plot) and nuclear (right plot) YAP1 staining and ERG status assessed by immunohistochemistry (IHC) and fluorescence *in situ* hybridisation (FISH). (**b,c**) Examples of cancer spots with (**b**) weak and (**c**) strong YAP1 staining in ERG negative prostate cancers. (**d,e**) Examples of cancer spots with (**d**) weak and (**e**) strong YAP1 staining in ERG positive prostate cancers.

Data on both ERG break-apart fluorescence *in-situ* hybridization (FISH) and ERG IHC were concordant in 95.5% of these 4,617 cancers with both FISH and IHC data. High cytoplasmic and nuclear YAP1 were both significantly linked to cancers with *TMPRSS2:ERG* rearrangement and ERG expression (Fig. 4). Because of these differences in YAP1 staining between ERG positive and ERG negative cancers, these subsets were also evaluated separately. Associations with tumour phenotype (Supplementary Tables S2 and S3) and PSA recurrence (Fig. 3c–e; p < 0.0019 each) were largely retained in these subgroups, both for nuclear and cytoplasmic staining.

### YAP1 and genomic deletion

Most deletions in prostate cancer are linked to either ERG negative cancers (i.e., deletions of 5q, 6q, 13q, 18q) or ERG positive cancers (i.e., deletions of 3p, 8p, 10q (*PTEN)*, 12q, 16q, 17q). Because YAP1 expression was also linked to a positive ERG status, it was not surprising to find that high nuclear and cytoplasmic YAP1 staining was linked to deletions of 8p, 10q (*PTEN*), 16q and 17p (p < 0.0001) if all cancers were jointly analysed (Supplementary Figs. S2, S3). However, a search for associations that do not depend on ERG must be carried out in separate subsets of cancers with ERG-positive and ERG-positive cancers. For both nuclear and cytoplasmic staining, these analyses revealed that YAP1 staining is linked to deletions of 8p and *PTEN* (10q) in both ERG positive and ERG negative cancers (p ≤ 0.0004 each, Supplementary Figs. S2, S3).

### YAP1, androgen receptor (AR) and tumour cell proliferation (Ki67 labelling index)

**Figure 5:**
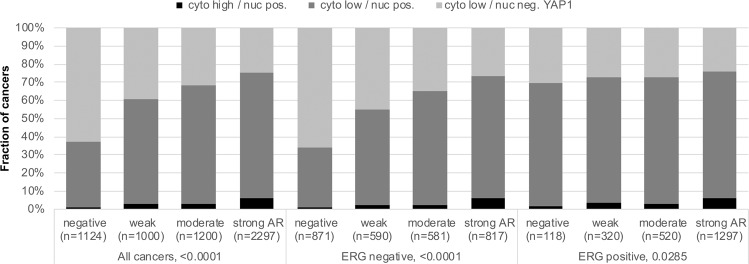
YAP1 and androgen receptor (AR). Correlation between different YAP1 staining patterns and AR expression levels in all cancers, ERG negative cancers and ERG positive cancers. “Cyto high” and “cyto low” includes cancers with moderate to strong (high) and negative to weak (low) cytoplasmic YAP staining. “Nuc pos” and “nuc neg” includes cancers with at least weak nuclear YAP1 staining (pos) and those lacking detectable nuclear YAP1 staining (neg).

Data on YAP1 and AR expression from 7,971 cancers showed a significant association between AR expression and cytoplasmic and nuclear YAP1 staining (Fig. 5 and Supplementary Fig. 4a,b). High YAP1 staining was also significantly linked to increased cell proliferation as measured by Ki67 labelling index. These associations were statistically significant for nuclear and cytoplasmatic staining in the analysis of all cancers (p < 0.0001) and in most subsets of cancers with identical Gleason score (Table 2, Supplementary Table S4, Supplementary Fig. 4c,d).

**Table 2:** Cytoplasmic YAP1 staining and Ki67 labelling index in all cancers, the ERG negative and positive subset.

Gleason	Cytoplasmic	All cancers			ERG negative cancers			ERG positive cancers		
Subset	YAP1	N	Mean ± SEM	P	n	Mean ± SEM	P	n	Mean ± SEM	P
**Total**	**Negative**	1073	1.9 ± 0.1		835	1.7 ± 0.1		213	2.8 ± 0.2	
	**Weak**	2104	2.7 ± 0.1	<0.0001	1186	2.7 ± 0.1	<0.0001	884	2.7 ± 0.1	0.1152
	**Moderate**	1988	3.1 ± 0.1		829	3.2 ± 0.1		1138	3 ± 0.1	
	**Strong**	171	3.4 ± 0.2		77	3.7 ± 0.3		90	3.1 ± 0.3	
**≤3+3**	**Negative**	301	1.6 ± 0.1		234	1.4 ± 0.1		53	2.4 ± 0.3	
	**Weak**	441	2.3 ± 0.1	<0.0001	204	2.2 ± 0.1	<0.0001	223	2.4 ± 0.1	0.9814
	**Moderate**	302	2.4 ± 0.1		95	2.5 ± 0.2		199	2.4 ± 0.1	
	**Strong**	13	2.1 ± 0.6		4	1.8 ± 1		9	2.2 ± 0.7	
**3+4**	**Negative**	556	1.8 ± 0.1		431	1.6 ± 0.1		117	2.5 ± 0.2	
	**Weak**	1156	2.5 ± 0.1	<0.0001	646	2.4 ± 0.1	<0.0001	494	2.6 ± 0.1	0.0225
	**Moderate**	1174	2.9 ± 0.1		452	2.9 ± 0.1		712	3 ± 0.1	
	**Strong**	101	2.7 ± 0.2		44	2.3 ± 0.3		55	3.1 ± 0.3	
**3+4 Tertiary 5**	**Negative**	36	2 ± 0.4		34	2 ± 0.4		2	2.5 ± 1.8	
	**Weak**	92	3.2 ± 0.3	0.0045	57	3.2 ± 0.3	0.0178	35	3.1 ± 0.4	0.3899
	**Moderate**	100	3.7 ± 0.2		55	3.7 ± 0.3		44	3.8 ± 0.4	
	**Strong**	2	4 ± 1.7		2	4 ± 1.7		0	0 ± 0	
**4+3**	**Negative**	95	2.7 ± 0.3		70	2.6 ± 0.4		23	3 ± 0.6	
	**Weak**	216	3.3 ± 0.2	0.1992	143	3.4 ± 0.3	0.1452	71	3.1 ± 0.3	0.9742
	**Moderate**	213	3.3 ± 0.2		108	3.4 ± 0.3		104	3.2 ± 0.3	
	**Strong**	26	4 ± 0.6		12	4.8 ± 1		13	2.9 ± 0.8	
**4+3 Tertiary 5**	**Negative**	41	2.9 ± 0.6		30	2.2 ± 0.7		10	4.9 ± 1.2	
	**Weak**	109	3.5 ± 0.4	0.0256	68	3.7 ± 0.5	0.0007	40	3.2 ± 0.6	0.46
	**Moderate**	115	4.3 ± 0.4		64	4.5 ± 0.5		50	4 ± 0.5	
	**Strong**	15	6 ± 1		6	9.2 ± 1.6		8	2.9 ± 1.3	
**≥4+4**	**Negative**	43	3.2 ± 0.7		35	2.5 ± 0.7		8	6 ± 1.9	
	**Weak**	88	4.6 ± 0.5	0.131	67	4.3 ± 0.5	0.016	20	5.7 ± 1.2	0.3989
	**Moderate**	84	4.5 ± 0.5		55	5 ± 0.6		29	3.4 ± 1	
	**Strong**	14	6.3 ± 1.2		9	6.6 ± 1.4		5	5.8 ± 2.4	

### Multivariable analysis

YAP1 predicted biochemical recurrence independent from established prognostic parameters (Table 3). The maximal univariate hazard ratio for PSA recurrence was 1.4 for strong versus negative nuclear YAP1 expression and 1.9 for cytoplasmic YAP1 expression. In the multivariable model, YAP1 expression together with the univariably significant preoperative variables (Gleason grade, clinical stage and PSA level) showed a maximal multivariate hazard ratio of 1.3 for nuclear and 1.6 for cytoplasmic YAP1 expression. These hazard ratios were below the values for the other established parameters.

**Table 3:** Multivariate hazard ratio (95% confidence interval) for biochemical relapse after prostatectomy for established risk factors and YAP1 expression in the preoperative model.

Variable	Category	Cytoplasmic	Nuclear
		n = 5,290	n = 8,553
Gleason grade biopsy	≥4 + 4 vs. ≤3 + 3	4.1 (3.6–4.7) ***	4.2 (3.7–4.7) ***
Preoperative PSA level	≥20 vs. <4	3.2 (2.7–3.9) ***	3.2 (2.7–3.9) ***
cT stage	T2c vs. T1c	2.2 (1.8–2.8) ***	2.1 (1.7–2.6) ***
YAP1 expression	Strong vs. negative	1.6 (1.3–1.9) ***	1.3 (1.1–1.5) **

Categories with the highest hazard ratio are shown for each variable ranked in decreasing order; *p ≤ 0.05, **p ≤ 0.001, ***p ≤ 0.0001

## Discussion

The results of our study demonstrate that YAP1 up regulation is linked to prostate cancer aggressiveness independently from established prognostic markers of the disease.

Often, both nuclear and cytoplasmic YAP1 staining of cancer cells did not unequivocally differ from the staining in normal prostate glands in our study. This fits well with earlier observations by Noh *et al*.^29^, who reported strongly positive basal cells but no significant differences between the variable staining present in the normal luminal cells and in the tumour cells of 188 prostate cancers. The absence of clear-cut YAP1 expression differences between normal and cancerous prostatic glands may also explain why other studies came to contradictory conclusions. Hu *et al*.^27^ described decreased YAP1 staining in tumour cells as compared to hyperplastic or normal glands in 66 cancers. Sheng *et al*.^30^ studied YAP1 expression in 62 tissue samples obtained from tumour, tumour adjacent normal tissue and benign prostatic hyperplasia, and reported YAP1 up regulation in cancers as compared to non-neoplastic cells.

The high number of tumours in this study allowed to find a clear-cut link between higher YAP1 staining levels and adverse tumour phenotype as well as unfavourable prognosis. The functional role of YAP1 is dependent on whether it locates to the cytoplasm or to the nucleus. To activate growth-control associated genes, YAP1 must translocate from the cytoplasm to the nucleus^4,31,32^. The separate analysis of both nuclear and cytoplasmic staining resulted in identical associations with tumour phenotype and patient prognosis, however. This fits well to a model where YAP1 activation during tumour progression includes up regulation, cytoplasmic accumulation and subsequent translocation to the nucleus. Functional studies have shown that nuclear translocation indicates activation of YAP1, leading to induction of growth-control associated genes^31,32^. In line with our results, Noh *et al*.^29^ found higher levels of both cytoplasmic and nuclear YAP1 in high Gleason grade than in low grade cancers and a significant link between high YAP1 expression and early biochemical recurrence in 188 tumours. Sheng *et al*.^30^ reported links between YAP1 overexpression and higher Gleason grade as well as lymph node involvement in 32 cancers. Zhang *et al*.^5^ described high level YAP1 expression in 13 castration resistant cancers but none or only low YAP1 staining in 7 hormone naïve cancers. Only one study on 66 cancers suggested associations between decreased YAP1 expression and high Gleason score^27^. That YAP1 up regulation (and not down regulation) promotes cancer progression fits also well to several studies from other tumour types^7,9,12,33–35^.

The molecular database collected during numerous studies in the past allowed a comparison of our YAP1 data with other relevant molecular alterations. About 50% of prostate cancers contain a gene fusion involving the androgen-regulated TMPRSS2 and the transcription factor *ERG*^36,37^. Androgen dependent ERG expression results in alteration of more than 1,500 genes in affected prostate epithelial cells^38^. The significant up-regulation of YAP1 in cancers having a *TMPRSS2:ERG* fusion is consistent with data showing that ERG can activate YAP1 dependent transcription and tumour development^23^. The significant association of YAP1 and androgen receptor expression fits well to its known interaction. AR and YAP1 have been shown to colocalize to the nucleus, and downregulation of YAP1 leads to suppression of AR target genes, suggesting that YAP1 is important for AR signalling^26^. A prognostic role of YAP1 expression was observed in subsets of both ERG positive and ERG negative cancers, althougth stronger in ERG negative tumours. This makes YAP1 expression analysis a universally applicable prognostic feature that is not dependent on a particular molecular prostate cancer subtype. In earlier studies using the same prostate cancer TMA, several molecular parameters had been identified that were only prognostic in either ERG positive^39,40^ or ERG negative cancers^41,42^.

Deletions of 3p13, 8p21, 10q23 (*PTEN*), 12q24, 16q24, and 17p13 are linked to ERG positive cancers and 5q21, 6q15, 13q14, and 18q21 to ERG negative cancers^41–47^. Only the 12q13 deletion is unrelated to the ERG status^40^. As YAP1 was strongly associated with a positive ERG status, it is not surprising that YAP1 was either positively or inversely related to most deletions when all cancers were jointly analyzed. However, the absence of an association between most of these deletions with YAP1 expression in ERG positive and ERG negative tumour subsets argues against a direct role of YAP1 for the control of genome integrity or double strand breakage repair. The particularly strong association between YAP1 expression and *PTEN* deletions fits well with earlier reports describing a direct interaction between PTEN and the Hippo pathway^24^. Inactivation of the PTEN lipid phosphatase terminated the MOB1-LATS1/2 interaction, decreased phosphorylation of YAP1, induced YAP1 nuclear translocation, and increased the synergism between YAP1 and TEAD, thus eventually inducing cell proliferation and migration^24^. The strong link between YAP1 expression and 8p deletions may partly be caused by the high rate of co-deletions of *8p* and *PTEN*. It is also possible that YAP1 has a relevant interaction with a specific 8p gene.

The present study proposes that the YAP1 protein level may be a weak, however useful, biomarker. Irrespective of whether nuclear or cytoplasmic YAP1 protein is measured, the prognostic impact of YAP1 staining was independent of conventional histo-morphological prognostic parameters. It is of note that all commonly used prognostic parameters in prostate cancer share major deficiencies. The Gleason grade, for example, suffers from very substantial interobserver variability, even between expert genitourinary pathologists^48^. The absence of a prognostic role of YAP1 expression in subests of cancers with identical quantitative Gleason grade highligths the power of the quantitative Gleason grading system^49,50^, although it is not universally applied and does not overcome all issues connected to interobserver variability in prostate cancer grading. The diagnosis of nodal metastasis is greatly dependent on the extent of surgery and the pathological work-up^51^. Accordingly, prognostic parameters are needed that are not necessarily statistically independent of established parameters but more reproducible and reliable than the established ones.

The polyclonal antibody against YAP1 that was used in this study strongly detected the protein in YAP1 overexpressing HeLa cells under identical experimental conditions as used for the TMA analysis. With the same protocol, non-transfected HeLa cells stained entirely negative, while HeLa cells transfected with YAP1’s paralog WWTR1 showed some faint staining with the YAP1 antibody. Given that YAP1 and WWTR1 share about 60% homology^52,53^, it cannot be excluded that we co-detected WWTR1 in addition to YAP1 at least in cancers with very high WWTR1 expression levels.

In summary, the data of this study show that YAP1 is a weak, however potentially useful, prognostic parameter in prostate cancer. The Hippo pathway and it’s downstream regulator YAP1 appear to play a similar important role in prostate cancer as it is known from many other solid cancer types. In the last 3 years, several Hippo pathway inhibititors that block the YAP-TEAD association have been developed and some show anti-tumour activity *in-vitro*^54^. Although still far from clinical application, such or similar substances may hold promizes for the therapy of many cancer types including early and advanced prostate cancers and prompt for diagnostic tests. For the future, we expect that panels composed of multiple antibodies, perhaps measured simultaneously by using multicolour fluorescence IHC, will be developed for prostate cancer prognosis assessment. Although analysis of the YAP1 protein alone had only a moderate – however independent – prognostic power in our study, it may represent a promising candidate for such a multiparametric prognostic approach.

## Materials and Methods

### Ethical statement

The study was approved by the Ethics Commission Hamburg, WF-049/09 and conducted in accordance with the Declaration of Helsinki. Informed consent has not been collected specifically for the patient samples included in this study. Usage of routinely archived formalin fixed leftover patient tissue samples for research purposes by the attending physician is approved by local laws and does not require written consent (HmbKHG, §12,1).

### Patients

The study involved a total of 17,747 patients who underwent radical prostatectomy between 1992 and 2012 (Department of Urology and the Martini Clinic at the University Medical Centre Hamburg-Eppendorf). Histopathological data included pT, pN, resection margin, Gleason grade and “quantitative” Gleason grading^49^. Follow-up was available for a total of 14,664 patients (median 48 months; range 1 to 276 months; Supplementary Table S5). Prostate specific antigen (PSA) recurrence was defined as a postoperative PSA of 0.2 ng/ml and increasing at first of appearance. The TMA contained a single 0.6 mm core from a tumour containing tissue block for each patient^55^.

### TMA database

The TMA was annotated with data from previous studies on Ki67 labelling Index (Ki67LI)^56^, androgen receptor (AR) expression^36^, and ERG protein expression^57^ that were assessed by means of immunohistochemistry (Supplementary Fig. 5). Genomic deletion of 3p13 (FOXP1)^43^, 5q21 (CHD1)^47^, 6q15 (MAP3K7)^42^, 8p21^58^, 10q23 (PTEN)^25^, 12p13 (CDKN1B)^45^, 12q24^36^, 13q14 (FOXO1, RB1)^41^, 16q24^44^, 17p13 (TP53)^46^ and 18q21^59^ as well as ERG rearrangement analysis^57^ was done by fluorescence *in situ* hybridization (FISH) using differentially labelled locus specific and centromere specific probes. Deletion was defined as less locus specific then centromere specific FISH probe signals in ≥60% of cancer cells.

### Generation of YPA1 and WWTR1 expressing control cells

Human cervix epithelial carcinoma (HeLa) cells were cultured in DMEM (Dulbecco’s Modified Eagles Medium), 10% fetal bovine serum (FBS) and 1% penicillin-streptomycin (P/S) at 37 °C and 5% CO_2_. Constructs encoding for YAP1 (cat. HG17690-UT, Sino Biological Inc., Wayne PA, USA) and WWTR1 (cat SC328639, Origene, Rockville, MD, USA) were each transfected into competent *Escherichia coli* cells (One Shot^TM^ Top10, ThermoFisher Scientific, Germany). After 24 h, amplified plasmid was extracted (cat # 740579, Macherey-Nagel, Düren, Germany) and transfected into 3 × 10^6^ HeLa cells (JetPEI DNA Transfection reagent, Polyplus-transfection S.A., Illkirch, France). Cells were grown for another 24 h, harvested, pelleted, fixed in 4% buffered formalin overnight and embedded in a paraffin block. Non-transfected HeLa cells were used as negative controls.

### Immunohistochemistry

Freshly cut TMA sections were stained in a single experiment. Slides were deparaffinized and antigen was retrieved by heat (121 °C, 5 min, pH 7.8 Tris-EDTA-citrate buffer). Primary antibody specific for YAP1 (rabbit polyclonal antibody from Cell Signaling Technology, Danvers, MA, USA; cat. #4912; dilution 1:50) was applied at 37 °C for 60 min. Bound antibody was then visualized using the EnVision Kit (Dako, Glostrup, Denmark) according to the manufacturer’s directions. YAP1 showed nuclear and cytoplasmic staining. Staining of YAP1 in the positive and negative control cells is shown in Supplementary Fig. 6. A trained pathologist manually scored nuclear and cytoplasmic staining in two separate rounds of analysis according to the following criteria: The staining intensity (0, 1+, 2+, 3+) as well as the fraction of stained cells was recorded for each tissue spot. The IHC results for cytoplasmic and nuclear staining were created from these two parameters as follows: Lack of any staining (intensity 0) was considered “negative”, 1+ staining in ≤70% of tumour cells or 2+ staining in ≤30% of tumour cells was considered “weak”, 2+ staining in ≤70% of tumour cells or 2+ staining in >30% but ≤70% of tumour cells or 3+ staining in ≤30% of tumour cells was considered “moderate”, and 2+ staining in >70% of tumour cells or 3+ staining in >30% of tumour cells was considered “strong”.

### Uni- and multivariable analysis

Uni- and multivariable hazard ratios for PSA recurrence were calculated for all categories. The variables significant in univariable analysis were included in the multivariable model. For comparison of the variables the categories with the maximal hazard ratio are given and ranked in decreasing order.

### Statistics

Contingency tables and the chi²-test were performed to search for associations between molecular parameters and tumour phenotype. Kaplan-Meier survival curves were calculated and compared by the log-rank test. Cox proportional hazards regression analysis was performed to test for independence and significance between pathological, molecular and clinical variables. Various models combining pre- and postoperative available parameters were calculated. JPM 12 was used for calculations (SAS Institute Inc., NC, USA).

### Ethical approval and informed consent

See above in Material and Methods.

## Supplementary information

Supplementary Information.

## Data Availability

Data are available from the corresponding author on reasonable request.
