# Supplementary material for: Up regulation of the Hippo signalling effector YAP1 is linked to early biochemical recurrence in prostate cancers
Source: Sci Rep. 2020 Jun 2;10:8916. doi: 10.1038/s41598-020-65772-w (PMC7265544; doi:10.1038/s41598-020-65772-w)
Supplement: Supplementary file 1 — Supplementary Information. [file 41598_2020_65772_MOESM1_ESM.docx]

**Supplementary material: Up regulation of the Hippo signalling effector YAP1 is linked to early biochemical recurrence in prostate cancers** Andreas Marx ^1,2,7^, Aljoscha Schumann^1,7^, Doris Höflmayer^1^, Elena Bady^1^, Claudia Hube-Magg^1^, Katharina Möller^1^, Maria Christina Tsourlakis^1^, Stefan Steurer^1^, Franziska Büscheck^1^, Till Eichenauer ^6^, Till S Clauditz^1^, Markus Graefen^4^, Ronald Simon^1,^*, Guido Sauter^1^, Jakob R Izbicki^3^, Hartwig Huland^4^, Hans Heinzer^4^, Alexander Haese^4^, Thorsten Schlomm^5^, Christian Bernreuther^1^, Patrick Lebok^1^ and Adam Polonski^1,3^

**Table S1. Nuclear YAP1 staining and prostate cancer phenotype**

|  | **Nuclear YAP1 staining (%)** | | | | | |
| --- | --- | --- | --- | --- | --- | --- |
|  | **N** | **Negative** | **Weak** | **Moderate** | **Strong** | ***P*** |
| **All cancers** | 9884 | 37.1 | 31.5 | 21.5 | 10.0 |  |
|  |  |  |  |  |  |  |
| **Tumour stage** | |  |  |  |  | <0.0001 |
| pT2 | 6188 | 40.6 | 31.3 | 19.2 | 8.9 |  |
| pT3a | 2330 | 34.1 | 31.2 | 23.6 | 11.2 |  |
| pT3b-pT4 | 1332 | 26.1 | 33.0 | 28.4 | 12.6 |  |
|  |  |  |  |  |  |  |
| **Gleason grade** | |  |  |  |  | <0.0001 |
| ≤3+3 | 1875 | 46.3 | 27.6 | 15.7 | 10.3 |  |
| 3+4 | 5311 | 37.7 | 31.9 | 20.8 | 9.7 |  |
| 3+4 Tert.5 | 436 | 36.5 | 36.0 | 21.3 | 6.2 |  |
| 4+3 | 1038 | 27.8 | 32.1 | 28.8 | 11.3 |  |
| 4+3 Tert.5 | 677 | 28.8 | 33.7 | 27.6 | 9.9 |  |
| ≥4+4 | 541 | 28.5 | 32.3 | 26.6 | 12.6 |  |
|  |  |  |  |  |  |  |
| 3+4 ≤5% | 1326 | 42.0 | 32.2 | 17.4 | 8.4 | <0.0001 |
| 3+4 6-10% | 1396 | 38.8 | 32.8 | 18.6 | 9.9 |  |
| 3+4 11-20% | 1183 | 36.5 | 30.4 | 24.1 | 9.0 |  |
| 3+4 21-30% | 626 | 33.9 | 33.2 | 24.8 | 8.1 |  |
| 3+4 31-49% | 529 | 35.2 | 31.4 | 22.5 | 11.0 |  |
| 4+3 50-60% | 446 | 28.7 | 33.6 | 26.5 | 11.2 |  |
| 4+3 61-80% | 393 | 26.0 | 33.6 | 30.5 | 9.9 |  |
| 4+3 >80% | 93 | 31.2 | 19.4 | 36.6 | 12.9 |  |
|  |  |  |  |  |  |  |
| **Lymph node metastasis** | | |  |  |  | <0.0001 |
| N0 | 5835 | 35.6 | 31.8 | 22.6 | 10.0 |  |
| N+ | 660 | 27.4 | 30.0 | 30.2 | 12.4 |  |
|  |  |  |  |  |  |  |
| **Preoperative PSA level (ng/ml)** | | |  |  |  | <0.0001 |
| <4 | 1139 | 34.6 | 26.3 | 24.8 | 14.3 |  |
| 4-10 | 5894 | 37.9 | 31.6 | 21.1 | 9.3 |  |
| 10-20 | 2052 | 37.4 | 33.5 | 20.2 | 9.0 |  |
| >20 | 735 | 34.1 | 33.5 | 22.3 | 10.1 |  |
|  |  |  |  |  |  |  |
| **Surgical margin** | |  |  |  |  | <0.0001 |
| Negative | 7776 | 38.3 | 31.2 | 20.7 | 9.9 |  |
| Positive | 2071 | 32.7 | 32.3 | 24.7 | 10.3 |  |

**Table S2. Nuclear YAP1 staining and Ki67 labelling index in all cancers, the ERG negative and positive subset**

| **Gleason** | **Nuclear** | **All cancers** | | |  | **ERG negative cancers** | | |  | **ERG positive cancers** | | |
| --- | --- | --- | --- | --- | --- | --- | --- | --- | --- | --- | --- | --- |
| **subset** | **YAP1** | N | Mean ±SEM | P |  | n | Mean ±SEM | P |  | n | Mean ±SEM | P |
| **Total** | **Negative** | 2066 | 2.3±0.1 |  |  | 1370 | 2.1±0.1 |  |  | 657 | 2.8±0.1 |  |
|  | **Weak** | 1840 | 2.9±0.1 | <0.0001 |  | 1001 | 3±0.1 | <0.0001 |  | 817 | 2.9±0.1 | 0.0788 |
|  | **Moderate** | 1097 | 3.1±0.1 |  |  | 435 | 3.1±0.1 |  |  | 641 | 3.1±0.1 |  |
|  | **Strong** | 483 | 3±0.1 |  |  | 175 | 3.2±0.2 |  |  | 297 | 2.9±0.2 |  |
|  |  |  |  |  |  |  |  |  |  |  |  |  |
| **≤3+3** | **Negative** | 519 | 1.8±0.1 |  |  | 312 | 1.4±0.1 |  |  | 187 | 2.5±0.2 |  |
|  | **Weak** | 318 | 2.3±0.1 | 0.0003 |  | 155 | 2.4±0.1 | <0.0001 |  | 153 | 2.3±0.2 | 0.6698 |
|  | **Moderate** | 174 | 2.4±0.1 |  |  | 57 | 2±0.2 |  |  | 111 | 2.6±0.2 |  |
|  | **Strong** | 87 | 2.3±0.2 |  |  | 28 | 2.2±0.3 |  |  | 55 | 2.5±0.3 |  |
|  |  |  |  |  |  |  |  |  |  |  |  |  |
| **3+4** | **Negative** | 1149 | 2.2±0.1 |  |  | 756 | 2±0.1 |  |  | 379 | 2.6±0.1 |  |
|  | **Weak** | 1036 | 2.7±0.1 | <0.0001 |  | 530 | 2.7±0.1 | <0.0001 |  | 498 | 2.8±0.1 | 0.0015 |
|  | **Moderate** | 619 | 3±0.1 |  |  | 232 | 2.7±0.1 |  |  | 377 | 3.2±0.1 |  |
|  | **Strong** | 266 | 2.7±0.1 |  |  | 89 | 2.8±0.2 |  |  | 171 | 2.7±0.2 |  |
|  |  |  |  |  |  |  |  |  |  |  |  |  |
| **3+4** | **Negative** | 72 | 2.6±0.3 |  |  | 58 | 2.5±0.3 |  |  | 14 | 3.2±0.7 |  |
| **Tertiary 5** | **Weak** | 86 | 3.4±0.3 | 0.0589 |  | 56 | 3.3±0.3 | 0.0443 |  | 29 | 3.6±0.5 | 0.9586 |
|  | **Moderate** | 48 | 3.8±0.4 |  |  | 19 | 4.2±0.6 |  |  | 29 | 3.5±0.5 |  |
|  | **Strong** | 19 | 3.8±0.6 |  |  | 11 | 3.8±0.8 |  |  | 8 | 3.8±0.9 |  |
|  |  |  |  |  |  |  |  |  |  |  |  |  |
| **4+3** | **Negative** | 175 | 3.3±0.2 |  |  | 128 | 3.5±0.3 |  |  | 43 | 3±0.4 |  |
|  | **Weak** | 201 | 2.9±0.2 | 0.0908 |  | 121 | 2.7±0.3 | 0.0551 |  | 79 | 3.2±0.3 | 0.8667 |
|  | **Moderate** | 140 | 3.5±0.3 |  |  | 69 | 3.8±0.4 |  |  | 69 | 3.2±0.3 |  |
|  | **Strong** | 58 | 4±0.4 |  |  | 23 | 4.4±0.7 |  |  | 34 | 3.5±0.5 |  |
|  |  |  |  |  |  |  |  |  |  |  |  |  |
| **4+3** | **Negative** | 82 | 3.6±0.4 |  |  | 59 | 3.3±0.5 |  |  | 22 | 4.8±0.8 |  |
| **Tertiary 5** | **Weak** | 101 | 3.9±0.4 | 0.8124 |  | 67 | 4.4±0.5 | 0.4457 |  | 33 | 3±0.6 | 0.2608 |
|  | **Moderate** | 68 | 4.1±0.5 |  |  | 29 | 4.3±0.8 |  |  | 37 | 3.8±0.6 |  |
|  | **Strong** | 26 | 3.3±0.8 |  |  | 10 | 4±1.3 |  |  | 16 | 2.9±0.9 |  |
|  |  |  |  |  |  |  |  |  |  |  |  |  |
| **≥4+4** | **Negative** | 68 | 3.9±0.6 |  |  | 56 | 3.1±0.6 |  |  | 12 | 7.8±1.8 |  |
|  | **Weak** | 97 | 5.3±0.5 | 0.188 |  | 72 | 5.3±0.5 | 0.0431 |  | 24 | 5.5±1.3 | 0.1793 |
|  | **Moderate** | 48 | 3.7±0.7 |  |  | 29 | 4.4±0.8 |  |  | 18 | 2.6±1.5 |  |
|  | **Strong** | 27 | 4.8±0.9 |  |  | 14 | 4.6±1.1 |  |  | 13 | 5±1.8 |  |

**Table S3. Cytoplasmic YAP1 staining and prostate cancer phenotype in the ERG negative subset**

|  | **Cytoplasmic YAP1 staining (%)** | | | | | |
| --- | --- | --- | --- | --- | --- | --- |
|  | **N** | **Negative** | **Weak** | **Moderate** | **Strong** | ***P*** |
| **All cancers** | 4311 | 26.6 | 41.2 | 29.5 | 2.8 |  |
|  |  |  |  |  |  |  |
| **Tumor stage** |  |  |  |  |  | <0.0001 |
| pT2 | 2815 | 27.9 | 42.1 | 27.7 | 2.3 |  |
| pT3a | 920 | 25.4 | 40.8 | 30.2 | 3.6 |  |
| pT3b-pT4 | 561 | 22.1 | 36.7 | 37.3 | 3.9 |  |
|  |  |  |  |  |  |  |
| **Gleason grade** |  |  |  |  |  | <0.0001 |
| ≤3+3 | 784 | 39.5 | 40.6 | 18.5 | 1.4 |  |
| 3+4 | 2303 | 25.8 | 41.9 | 29.5 | 2.8 |  |
| 3+4 Tertiary 5 | 195 | 22.1 | 38.5 | 38.5 | 1.0 |  |
| 4+3 | 492 | 19.7 | 42.7 | 34.8 | 2.8 |  |
| 4+3 Tertiary 5 | 273 | 16.8 | 40.3 | 38.1 | 4.8 |  |
| ≥4+4 | 261 | 21.1 | 36.0 | 36.8 | 6.1 |  |
|  |  |  |  |  |  |  |
| 3+4 ≤5% | 600 | 28.5 | 41.0 | 27.5 | 3.0 | <0.0001 |
| 3+4 6-10% | 603 | 23.5 | 44.1 | 29.2 | 3.2 |  |
| 3+4 11-20% | 517 | 27.9 | 42.2 | 28.0 | 1.9 |  |
| 3+4 21-30% | 263 | 22.1 | 41.4 | 34.2 | 2.3 |  |
| 3+4 31-49% | 241 | 24.5 | 38.2 | 32.8 | 4.6 |  |
| 4+3 50-60% | 203 | 21.2 | 44.3 | 32.5 | 2.0 |  |
| 4+3 61-80% | 192 | 16.7 | 41.7 | 38.0 | 3.6 |  |
| 4+3 >80% | 54 | 16.7 | 38.9 | 40.7 | 3.7 |  |
|  |  |  |  |  |  |  |
| **Lymph node metastasis** | |  |  |  |  | 0.0241 |
| N0 | 2543 | 23.3 | 41.8 | 31.7 | 3.3 |  |
| N+ | 266 | 23.7 | 33.8 | 39.5 | 3.0 |  |
|  |  |  |  |  |  |  |
| **Preoperative PSA level (ng/ml)** | | |  |  |  | 0.0576 |
| <4 | 434 | 22.4 | 44.2 | 30.0 | 3.5 |  |
| 4-10 | 2527 | 26.1 | 40.9 | 30.3 | 2.8 |  |
| 10-20 | 958 | 28.9 | 42.1 | 26.9 | 2.1 |  |
| >20 | 365 | 29.6 | 37.0 | 29.3 | 4.1 |  |
|  |  |  |  |  |  |  |
| **Surgical margin** |  |  |  |  |  | 0.5274 |
| Negative | 3389 | 26.6 | 41.6 | 29.1 | 2.7 |  |
| Positive | 907 | 26.5 | 39.4 | 31.1 | 3.1 |  |

**Table S4. Cytoplasmic YAP1 staining and prostate cancer phenotype in the ERG positive subset**

|  | **Cytoplasmic YAP1 staining (%)** | | | | | |
| --- | --- | --- | --- | --- | --- | --- |
|  | **N** | **Negative** | **Weak** | **Moderate** | **Strong** | ***P*** |
| **All cancers** | 3346 | 9.2 | 37.3 | 49.2 | 4.3 |  |
|  |  |  |  |  |  |  |
| **Tumor stage** |  |  |  |  |  | <0.0001 |
| pT2 | 1956 | 8.5 | 39.9 | 48.3 | 3.2 |  |
| pT3a | 914 | 9.1 | 33.9 | 51.2 | 5.8 |  |
| pT3b-pT4 | 466 | 12.2 | 32.4 | 49.1 | 6.2 |  |
|  |  |  |  |  |  |  |
| **Gleason grade** |  |  |  |  |  | <0.0001 |
| ≤3+3 | 648 | 11.0 | 45.1 | 41.7 | 2.3 |  |
| 3+4 | 1925 | 8.4 | 35.8 | 51.5 | 4.2 |  |
| 3+4 Tertiary 5 | 111 | 5.4 | 43.2 | 51.4 | 0.0 |  |
| 4+3 | 346 | 10.1 | 31.8 | 50.9 | 7.2 |  |
| 4+3 Tertiary 5 | 198 | 9.1 | 34.3 | 48.5 | 8.1 |  |
| ≥4+4 | 117 | 13.7 | 33.3 | 46.2 | 6.8 |  |
|  |  |  |  |  |  |  |
| 3+4 ≤5% | 482 | 8.3 | 39.2 | 50.0 | 2.5 | <0.0001 |
| 3+4 6-10% | 503 | 7.6 | 36.0 | 52.1 | 4.4 |  |
| 3+4 11-20% | 410 | 8.3 | 32.0 | 54.6 | 5.1 |  |
| 3+4 21-30% | 244 | 8.6 | 39.3 | 46.7 | 5.3 |  |
| 3+4 31-49% | 181 | 6.6 | 31.5 | 56.9 | 5.0 |  |
| 4+3 50-60% | 152 | 9.2 | 32.9 | 53.3 | 4.6 |  |
| 4+3 61-80% | 129 | 8.5 | 34.9 | 46.5 | 10.1 |  |
| 4+3 >80% | 28 | 10.7 | 10.7 | 75.0 | 3.6 |  |
|  |  |  |  |  |  |  |
| **Lymph node metastasis** | |  |  |  |  | 0.6949 |
| N0 | 1936 | 9.3 | 35.7 | 50.1 | 4.9 |  |
| N+ | 222 | 11.7 | 34.2 | 48.6 | 5.4 |  |
|  |  |  |  |  |  |  |
| **Preoperative PSA level (ng/ml)** | | |  |  |  | 0.0728 |
| <4 | 464 | 8.0 | 31.7 | 55.4 | 5.0 |  |
| 4-10 | 2053 | 9.1 | 37.7 | 49.1 | 4.1 |  |
| 10-20 | 594 | 10.3 | 40.4 | 44.3 | 5.1 |  |
| >20 | 212 | 10.4 | 35.8 | 50.0 | 3.8 |  |
|  |  |  |  |  |  |  |
| **Surgical margin** |  |  |  |  |  | 0.3403 |
| Negative | 2604 | 8.9 | 37.5 | 49.5 | 4.1 |  |
| Positive | 731 | 10.4 | 36.3 | 48.2 | 5.2 |  |

**Table S5** Composition of the prostate cancer prognosis tissue microarray.

|  | **No. of patients (%)** | |
| --- | --- | --- |
|  | **Study cohort on TMA*** | **Biochemical relapse among categories** |
| **Follow-up** |  |  |
| n | 14464 | 3612 (25%) |
| Mean / Median (month) | 56 / 48 | - |
| **Age (y)** |  |  |
| ≤50 | 433 | 66 (15.2%) |
| 51-59 | 4341 | 839 (19.3%) |
| 60-69 | 9977 | 2073 (20.8%) |
| ≥70 | 2936 | 634 (21.6%) |
| **Pretreatment PSA (ng/ml)** | |  |
| <4 | 2225 | 313 (14.1%) |
| 4-10 | 10,520 | 1696 (16.1%) |
| 10-20 | 3662 | 1043 (28.5%) |
| >20 | 123 | 545 (44.3%) |
| **pT stage (AJCC 2002)** |  |  |
| pT2 | 11,518 | 1212 (10.5%) |
| pT3a | 3842 | 1121 (29.2%) |
| pT3b | 2233 | 1213 (54.3%) |
| pT4 | 85 | 63 (74.1%) |
| **Gleason grade** |  |  |
| ≤3+3 | 3570 | 264 (7.4%) |
| 3+4 | 9336 | 1436 (15.4%) |
| 3+4 Tert.5 | 1697 | 165 (9.7%) |
| 4+3 | 2903 | 683 (23.5%) |
| 4+3 Tert.5 | 1187 | 487 (41%) |
| ≥4+4 | 999 | 531 (53.2%) |
| **pN stage** |  |  |
| pN0 | 10,636 | 2243 (21.1%) |
| pN+ | 1255 | 700 (55.8%) |
| **Surgical margin** |  |  |
| Negative | 14,297 | 2307 (16.1%) |
| Positive | 3388 | 1304 (38.5%) |

* Numbers do not always add up to 17,747 in the different categories because of cases with missing data. Abbreviation: AJCC, American Joint Committee on Cancer.

**Supplementary Figure S1.** YAP staining in prostate cancers with a) Gleason score 3+3, b) Gleason score 3+4, c) Gleason score 4+3 and d) Gleason score ≥4+4. The asterisk (*) indicates normal prostate glands, the arrow indicates the basal cells. Note the strong YAP1 staining of basal cells.

**Supplementary Figure S2.** Cytoplasmic YAP1 staining and genomic deletions in all cancers, the ERG negative and positive subset.

**Supplementary Figure S3.** Nuclear YAP1 staining and genomic deletions in all cancers, the ERG negative and positive subset.

**Supplementary Figure S4.** YAP1 immunostaining in prostate cancers with a) low (negative/weak) androgen receptor (AR) staining, b) high (moderate/strong) androgen receptor (AR) staining, c) low (≤1%) Ki67 labeling index (LI) and d) high (<3%) Ki67LI.


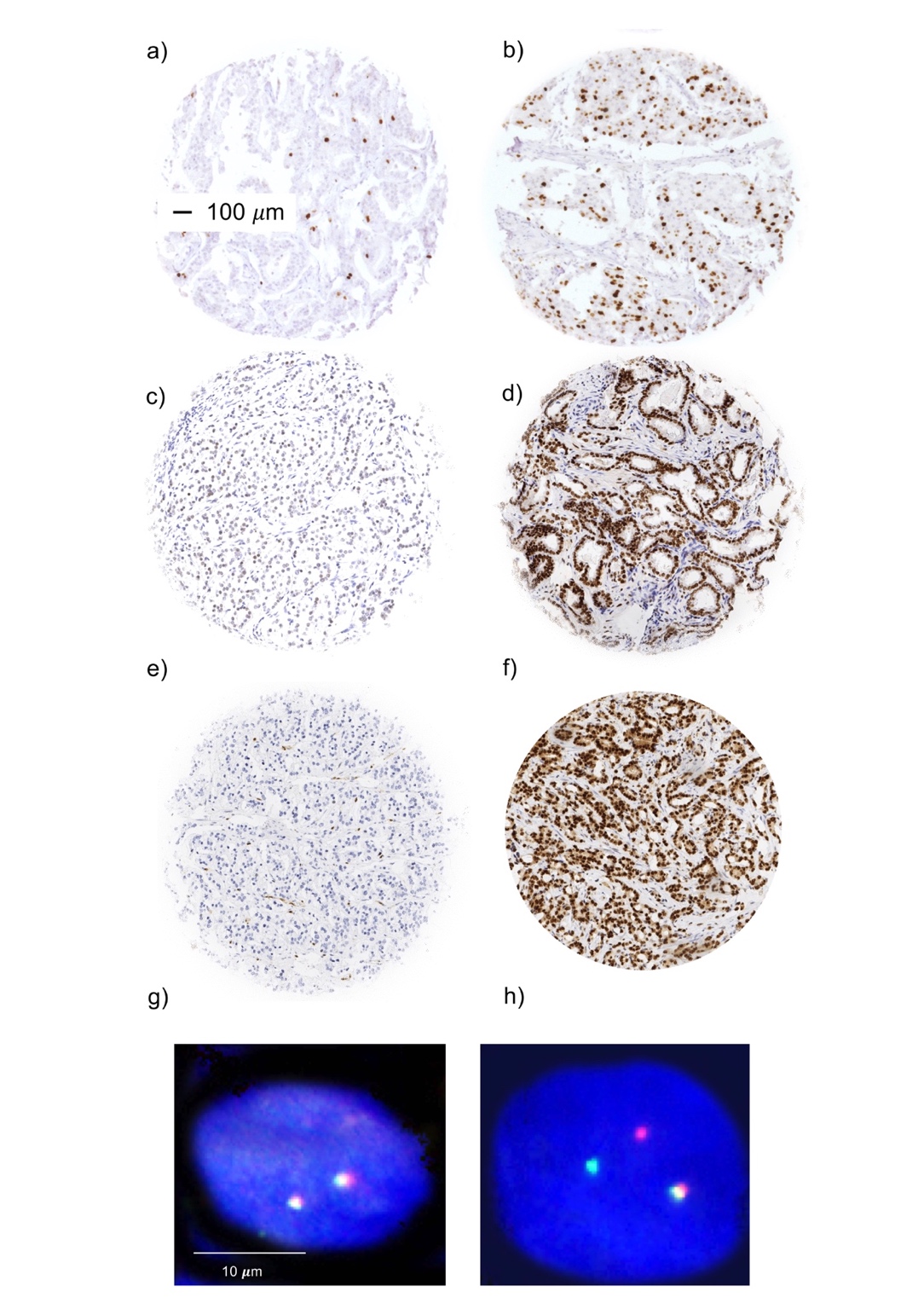


**Supplementary Figure S5.** Ki67 immunostaining in prostate cancers with a) low and b) high staining (antibody: Anti-Ki-67/MIB1, clone Ki-67P, dilution 1:400, Dianova) ^1^

Androgen receptor (AR) staining with negative staining and c) strong staining d. ) (antibody: Anti-Androgen Receptor, clone 2F12, dilution 1:10, Novocastra) ^2^

ERG immunostaining in prostate cancer with e) negative staining and f) positive (ERG IHC using antibody ERG (clone EPR3864, dilution 1:450, Epitomics) ^2^

ERG FISH in prostate cancer g) ERG normal indicate by two fusion signals and h) ERG gene breakage indicate by one red/green fusion signal and one split signal. ^2^

1 Minner, S. *et al.* Low level HER2 overexpression is associated with rapid tumor cell proliferation and poor prognosis in prostate cancer. *Clin Cancer Res* **16**, 1553-1560, doi:10.1158/1078-0432.CCR-09-2546 (2010).

2 Minner, S. *et al.* ERG status is unrelated to PSA recurrence in radically operated prostate cancer in the absence of antihormonal therapy. *Clin Cancer Res* **17**, 5878-5888, doi:10.1158/1078-0432.CCR-11-1251 (2011).

**Supplementary Figure S6.** YAP1 immunohistochemistry in a) non-transfected HeLa cells (negative control), b) HeLa cells transfected with a YAP1 expression (positive control) and c) HeLa cells transfected with a WWTR1 expression vector.
